# Hybrid Organic–Inorganic Perovskite Halide Materials for Photovoltaics towards Their Commercialization

**DOI:** 10.3390/polym14051059

**Published:** 2022-03-07

**Authors:** Luke Jonathan, Lina Jaya Diguna, Omnia Samy, Muqoyyanah Muqoyyanah, Suriani Abu Bakar, Muhammad Danang Birowosuto, Amine El Moutaouakil

**Affiliations:** 1 Department of Renewable Energy Engineering, Prasetiya Mulya University, Kavling Edutown I.1, Jl. BSD Raya Utama, BSD City, Tangerang 15339, Indonesia; 23301810001@student.prasetiyamulya.ac.id (L.J.); lina.diguna@prasetiyamulya.ac.id (L.J.D.); 2 Department of Electrical and Communication Engineering, College of Engineering, United Arab Emirates University, Al Ain P.O. Box 15551, United Arab Emirates; 202090009@uaeu.ac.ae; 3 Department of Physics, Faculty of Science and Mathematics, Universiti Pendidikan Sultan Idris, Tanjung Malim 35900, Malaysia; anna.physics87@gmail.com (M.M.); suriani@fsmt.upsi.edu.my (S.A.B.); 4 Łukasiewicz Research Network—PORT Polish Center for Technology Development, Stabłowicka 147, 54-066 Wrocław, Poland

**Keywords:** power conversion efficiency, hybrid perovskite, tandem structure, photovoltaics, commercialization

## Abstract

Hybrid organic–inorganic perovskite (HOIP) photovoltaics have emerged as a promising new technology for the next generation of photovoltaics since their first development 10 years ago, and show a high-power conversion efficiency (PCE) of about 29.3%. The power-conversion efficiency of these perovskite photovoltaics depends on the base materials used in their development, and methylammonium lead iodide is generally used as the main component. Perovskite materials have been further explored to increase their efficiency, as they are cheaper and easier to fabricate than silicon photovoltaics, which will lead to better commercialization. Even with these advantages, perovskite photovoltaics have a few drawbacks, such as their stability when in contact with heat and humidity, which pales in comparison to the 25-year stability of silicon, even with improvements are made when exploring new materials. To expand the benefits and address the drawbacks of perovskite photovoltaics, perovskite–silicon tandem photovoltaics have been suggested as a solution in the commercialization of perovskite photovoltaics. This tandem photovoltaic results in an increased PCE value by presenting a better total absorption wavelength for both perovskite and silicon photovoltaics. In this work, we summarized the advances in HOIP photovoltaics in the contact of new material developments, enhanced device fabrication, and innovative approaches to the commercialization of large-scale devices.

## 1. Introduction

Nowadays, silicon photovoltaics (PVs) have successfully achieved a high power conversion efficiency (PCE) of 26.7% [1] and this nearly approaches the theoretical limit value (29.4%) [2], and has led the PV sale sector, with more than 90% of the market share [3]. However, its numerous drawbacks, such as the scarcity of its pure state, the requirement of high energy to separate the bonded oxygen in silicon dioxide, and their PCE stagnancy, which has kept it in the range of 25% for over 15 years, have limited silicon PVs for further development. Perovskite PVs with a higher PCE are a promising PV replacement in overcoming the limitations of silicon PVs. After a decade of development, a high PCE value of 29.3% [4] has been achieved by perovskite PVs, and this value is similar to the theoretical limit value for silicon PVs. Other advantages offered by perovskite PVs include its capability in absorbing the visible spectrum, simplicity and cost-effective production (about $2.5/cell) [5,6].

The meta-analysis study presented in Ref. [7] provides a comprehensive view of the total costs of different PV materials, where CdTe was found to have the least capital and lifecycle costs among the PVs. Another analysis, specifically on perovskite tandem costs, in [8] emphasizes the commercial potential of PV perovskite tandem cells. The structure and light absorption of the perovskite layer, which depends on the energy level and carrier transport properties, will affect the PV’s PCE value. Therefore, most research has been focused on optimizing the morphology, energy level, and conductivity of perovskite PVs. Enhancing the structure, reducing defects, and increasing the grain size are several ways to improve the performance of perovskite PVs. These efforts have definitely been affected by the materials used in the synthesis process, and the selection of device fabrication methods [9,10].

With more research being focused on perovskites, HOIPs have been a focus since their emergence, and have shown numerous great characteristics, such as easy growth due to their cubic structures [9,10], a wide absorption range compatible with the solar spectrum [11], and a low exciton binding energy with a long carrier diffusion length [12]. All of these examples make HOIPs promising materials for emerging photovoltaic technology.

As an example of a HOIP, methylammonium lead halide (MAPbX_3_, X = Cl^−^, Br^−^, or I^−^) was the first material used for perovskite PV fabrication and resulted in a 3.8% PCE value, in 2009 [13]. In more than 10 years, this PCE value has increased to 29.8% by applying a smaller active area of 1 cm^2^ and by continuously focusing on improving operational stability [4]. This rapid progress has triggered interest in transferring existing technology, from small-area to large-area perovskite, which is necessary for industrial expansion [14]. To meet the requirements of a high quality and large-area uniformity for perovskite films, several deposition methods, based on solution, vacuum [15,16,17], and solution–vacuum hybrid processes, have been developed and optimized. Recent works have been based on solution processes, such as spin-coating [18], spray-coating [19,20], blade coating [21,22], slot-die coating [23,24], softcover [25,26,27], and screen printing [28,29,30]. However, even with several achievements in terms of grain size and increases in efficiencies through different fabrication method, these are still not enough for successful commercialization of perovskite PVs due to the remaining challenges that need to be solved. The primary challenge here is to achieve long lifetimes with a good stability at the module level [31]. Although much progress has been made, it is still challenging for perovskite PVs to reach the most popular international standards (IEC61215:2016) for mature PV technologies.

This challenge has triggered further studies on joining the structure of perovskite and silicon PVs, so-called tandem PVs. This effort was undertaken in order to improve PV performance through a combined system that can efficiently absorb solar radiation in both the visible and the near-infrared region [32]. In addition, due to the possibility of bandgap tuning, the perovskites themselves can be combined into an all-perovskite tandem photovoltaic, with both the top and bottom cells using a perovskite absorber. All of these efforts are still very young technologies, but enable remarkable PCEs, close to—or even above—those of the best single-junction cells [33,34]. This potential has been recognized, not only by various research institutes, but also by several start-up companies, such as Oxford PV, Swift Solar, and Tandem PV, who have developed their own perovskite PVs for commercialization.

Previous reviews discussed the effectiveness of hole transport and molecular materials in PV cells, where we can obtain cells with PCEs of up to 25% [35,36]. Other detailed reviews dedicated to hybrid organic–inorganic halide perovskites have emphasized that HOIPs are high-quality materials for PVs [37,38]. Finally, a recent review discussed different perovskite fabrication methods to develop large-area PVs with moderate-to-high PCEs [39]. In this work, we present a review that summarized advances in perovskite to perovskite–silicon tandem PVs, as this leads to a better commercialization. Advancements in materials to improve characteristics, leading to large-scale devices that are prepared for commercialization, are discussed in detail. Finally, we also highlight future research directions to achieve advances in extensive commercialization.

## 2. Materials and Fabrication Methods

### 2.1. Materials

**Figure 1:**
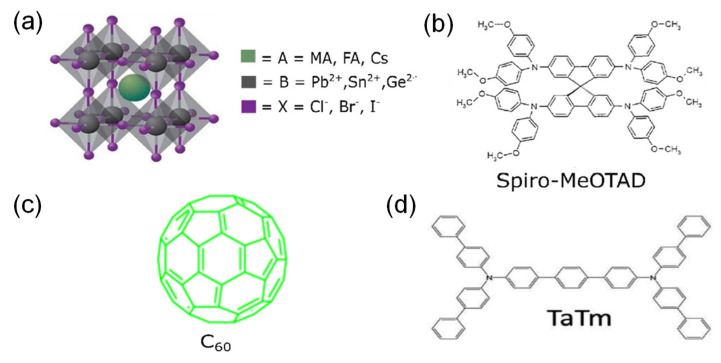
Common materials used in perovskite PVs: (**a**) crystal structure of MAPbI_3_, MAPbBr_3_, FAPbI_3_, FAPbBr_3_ [52]. Reproduced with permission from Ref. [52]. Copyright 2016 Advanced Science. (**b**) Hole transport layer (HTL) from Spiro−MeOTAD [53]. Reproduced with permission from Ref. [53]. Copyright 2017 Advanced Materials Interfaces. (**c**) HTL from TaTm [50]. Reproduced with permission from Ref. [50]. Copyright 2020 Frontiers in Chemistry. (**d**) Electron transport layer (ETL) from C60 [54]. Reproduced with permission from Ref. [54]. Copyright 2019 Scientific Reports.

Perovskite is a type of PV that includes perovskite-structured compounds that use tin or lead halides to act as the base material for a light-harvesting active layer [40]. The material of each perovskite differs, as they can be hybrids of organic and inorganic materials. All hybrid organic–inorganic perovskite (HOIP) materials have the general chemical formula of ABX_3_, as illustrated in Figure 1a. HOIP PVs are typically made using an organic/inorganic cation (A = methylammonium (MA) CH_3_NH_3_^+^, formamidinium (FA) CH_3_(NH_2_)_2_^+^) [41], a divalent cation (B = Pb^2+^ or Sn^2+^) [42], and an anion (X = Cl^−^, Br^−^, or I^−^). The perovskite material, ABX_3_, is sandwiched between electron transport layers (ETLs) and hole transport layers (HTLs), which are light absorbers. ETLs and HTLs have a pivotal role in charge transportation, separation, and recombination [43]. Examples of HTLs are shown in Figure 1b,c. Although a HOIP is a very attractive option for commercial applications, because this type of cell is very cheaply produced during a scale-up process [44], ETLs and HTLs face some challenges in terms of charge transfer [45]. One HTL used with a HOIP, such as spiro-OMeTAD (Figure 1b) has a low hole mobility and conductivity, but it does improve the optical and electrical properties of the HOIP [46]. Another HTL, like N4,N4′,N4″,N4′″-tetra([1,1-biphenyl]-4-yl)-[1,1:4,1-terphenyl]-4,4′-diamine (TaTm) in Figure 1c, allows efficient charge extraction, though there is a large misalignment of the highest occupied molecular orbit with the valence band of the HOIP [47]. The alignment of the valence and conduction bands of the perovskites with the transport layers of the HTLs and ETLs, respectively, is a critical factor for charge transfer and extraction. Some efforts have moved towards finding new materials for ETLs and HTLs, while others have investigated doping the layers to acquire enhanced properties [48]. Doping requires additives and solvents that may be hazardous, toxic, or harmful to the environment. Dopant-free structures have been developed to obtain better PCEs and stability without any additives [49,50]. An example for use as an ETL is C60 (Figure 1d) and it can achieve both the high stabilized power output and long-term operational stability of HOIP solar cells [51].

Low-dimensional perovskites, such as quasi two-dimensional (2D) perovskites, are known for their stability, but they have a lower performance than that of three-dimensional (3D) ones [55]. They have unique optical and charge transport properties, but also a high open circuit voltage (V_oc_) loss. These 2D halides can be used for photodetectors, lasers, resistive, solar cells, and LEDs. The 1D halide perovskites can be used in photodetectors and lasers; however, their use is challenging in other devices due to their rough structure and incomplete surface coverage, which result in a degraded performance. The 0D halide perovskite, or quantum dots, can be used in solar cells as they have a high photoluminescence quantum yield (up to 90%); however, their performance lags behind that of 3D halides because of their random orientation and the excess organic ligands on their surfaces, which reduce carrier mobility [56,57].

#### 2.1.1. Methylammonium Lead Triiodide (MAPbI_3_)

Metal halide perovskite (MAPbX_3_, X = Cl^−^, Br^−^, or I^−^) is the initial material used for perovskite PV fabrication and has a direct bandgap energy. The valence band is dominated by the p orbitals of the iodide, and the conduction band consists of Pb p orbitals; therefore, the transitions are p-to-p orbitals. Furthermore, the bandgap can be modified by varying A, B, and X. MAPbI_3_ is an example of such a material and possesses several advantages, such as having an absorption of 800 nm, a direct bandgap of 1.57 eV [13], a large absorption coefficient of 1.5 × 10^4^ cm^−1^ at 550 nm [58], a low-exciton binding energy of less than 10 meV [58], a very high charge carrier mobility of 66 cm^2^/Vs, a large electron and hole diffusion length of over 1 μm, and potentially reaches over 100 μm [59], thus it is suitable to be applied as a perovskite material.

#### 2.1.2. Formamidinium Lead Triiodide (FAPbI_3_)

Formamidinium lead triiodide (FAPbI_3_) has a bandgap energy of 1.48 eV near the optimal bandgap (1.34 eV), which is ideal for theoretical maximum device efficiency. Moreover, its excellent thermal- and photostability, in the form of black-phase FAPbI_3_ (α-FAPbI_3_), meets the requirements of perovskite PV devices. The earlier-reported FAPbI_3_-based perovskite PVs have had their advantages of having a large charge-carrier diffusion and high short-circuit currents established [60]. Compared to MAPbI_3_, FAPbI_3_ is more thermally stable due to the variations in A-site cations, as an FA cation has a greater thermal stability compared to an MA cation [61]. As methylammonium cations are volatile, the MA^+^ component will experience fragmentation with increased temperature, which leads to the quick decomposition of the MAPbI_3_ crystal structure [62]. Moreover, the activation energies for thermal degradation of FAPbI_3_ (115 ± 3 kj mol^−1^) are higher than those of MAPbI_3_ (93 ± 8 kj mol^−1^), so FAPbI_3_ has a better resistance to thermal decomposition [63]. In terms of humidity stability, both MAPbI_3_ and FAPbI_3_ can chemically decompose into lead iodide (PbI_2_) under high moisture conditions; however, when exposed to moisture, the FAPbI_3_ perovskite will undergo phase transition into an undesirable yellow non-perovskite (δ-FAPbI_3_) [64,65]. Furthermore, the deposition and fabrication methods of FAPbI_3_ films have difficulties in crystallizing the black perovskite phase.

#### 2.1.3. Mixed-Cation Perovskite

Since there are concerns with MAPbI_3_’s structural phase transitions and thermal stability, a combination of both FAPbI_3_ and MAPbI_3_ have been introduced with different ratios. With the perovskite composition of FA_1−x_MA_x_PbI_3_, when x = 0–1, it shows different SEM characteristics. Based on the ratio of FAPbI_3_ and MAPbI_3_, the crystal structure shows some cracks that differ, based on the x composition. With FA_0.8_MA_0.2_PbI_3_, there are several needle-like structures that can be obtained from the low MA contents, which lead to the formation of the δ-FAPbI_3_ phase, while FA_0.4_MA_0.6_PbI_3_ shows a better crystal structure with a lack of needle-like structures due to the increase in MA content, which results in the stabilization of α-FAPbI_3_ [66]. The results showed an improvement in the optoelectronic properties and in the stability [67,68,69]. This was due to the larger ionic radius provided by FA and the dual-ammonia group that inhibits ion movement in the space of the PbI_6_ octahedral [60]. However, a decrement of the open-circuit voltage (V_oc_) value was observed when mixing FA and MA (with a V_oc_ of about 1.02 V) [70,71,72]. A slightly higher V_oc_ value of 1.03 V was then achieved by adding additional layers through surface passivation [69,73]. Moreover, a FAMA mixed cation perovskite will tend to form an undesirable yellow perovskite, so CsI is introduced into the FAMA mixed cation perovskite to guarantee the formation of black phase perovskite PV [67,73,74]. With the introduction of a third cation, in this case Cs with the mixture of MA and FA cation, it is recognized as a triple cation perovskite.

### 2.2. Fabrication Methods

The aim of PV devices is to generate high and efficient power to be used in large systems. However, controlling the morphology of perovskite is a difficulty in scaling large-area PVs, especially as this situation is not present in small-area PVs. Therefore, the method of fabrication must be carefully selected. The reported scalable deposition methods for perovskite PVs are solution-based and vapor-based deposition techniques.

The fabrication method is usually divided into one- or two-step depositions. In one-step deposition, both precursors’ solutions are used, while for two-step deposition separating the layer deposition and the precursor solution to produce highly uniform and defect-free layers with a great morphology is performed. The layers obtained through one-step deposition has defects and, therefore, a higher recombination rate. Thus, two-step deposition offers better morphology control than one-step deposition through its deposition process [75].

#### 2.2.1. Solution Processing-Based Method

Solution processing-based methods are the common methods for perovskite deposition. Various methods derived from solution processing include spin-coating, dip-coating, doctor blade, spray-coating, ink-jet printing, screen printing, drop-casting, and slot die coating.

#### 2.2.2. Spin-Coating

**Figure 2:**
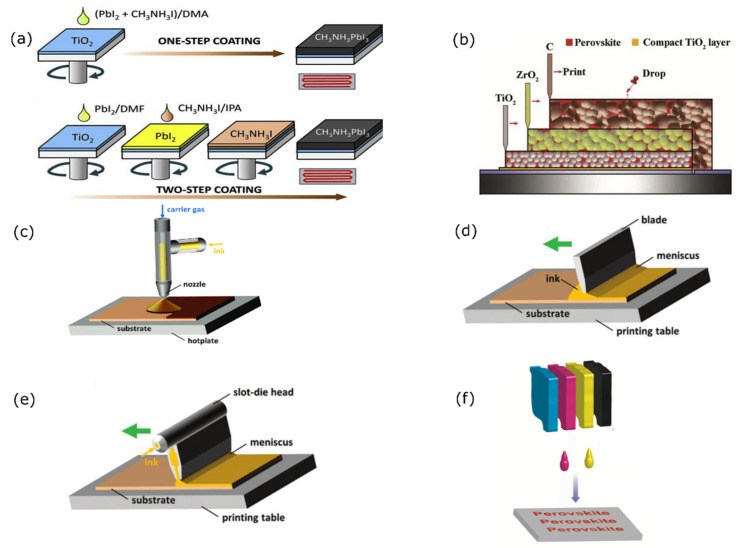
Different solution processing-based method for perovskite PV fabrication: (**a**) spin-coating [88], (**b**) drop-casting [88]. Reproduced with permission from Ref. [88]. Copyright 2017 Journal of Materiomics. (**c**) Spray-coating, (**d**) doctor blade, and (**e**) slot-die coating [89]. Reproduced with permission from Ref. [89]. Copyright 2019 Advanced Materials. (**f**) Ink-jet printing [90]. Reproduced with permission from Ref. [90]. Copyright 2016 Hal.

Spin-coating is the simplest and most cost-effective method derived from solution processing methods. This method is done by spin-coating a precursor on a substrate followed by annealing the thin film to obtain a crystallized layer of perovskite, as shown in Figure 2a. The layer thickness can be optimized by changing the spin speed, acceleration, and spin-coating time. The advantage of using this technique is the good level of reproducibility and the morphology obtained for small-scale areas; however, the long processing duration and material waste limit this method for application for large-scale area perovskite fabrication [76].

#### 2.2.3. Drop-Casting

Drop-casting is considered a cheap technique to produce PVs. The technique is based on dropping a volatile solvent on a substrate before it is evaporated and dried, as shown in Figure 2b. Layer thickness is dependent on the rate of evaporation, the drying process, and the volume and concentration of the solvent used for the dispersion. The wasted material in the case of drop-casting is less than that of spin-coating, which is counted as an advantage compared to the other method. However, the drawbacks of this method relate to the difficulty in controlling the layer thickness, which results is a non-uniform film formation which is thus ineffective for large-scale areas [77].

#### 2.2.4. Spray-Coating

Spray-coating is a highly scalable deposition method for large-scale area perovskite PV fabrication. This technique has several advantages, such as rapid film deposition, as the spray head can move across a substrate at more than 5 m per minute, as shown in Figure 2c. Other advantages include the low cost of processing, and the capability for deposition on flexible and glass-based substrates for use in large-scale perovskite photovoltaics. Moreover, spray-coated films are characterized by their high thermal stability and they have better optoelectronic properties compared to spin-coated films due to a better charge transfer capability and a longer minority carrier lifetime [78]. However, the drawback of this method is the difficulty in achieving fully homogeneous layers due to there being unpredictable characteristics in the film. This problem occurs when different droplet sizes leave different patches sizes during the drying process, thus increasing the series resistance that affects the performance. These problems can be solved using a modified spraying technique, such as pen spray [79], electrostatic spray-coating [80], pulsed spray coating [81], and ultrasonic spray-coating [82].

#### 2.2.5. Doctor Blade

Doctor blade is a simple coating system that uses a blade coater applicator—in this method, the height of the blade is adjusted depending on the substrate surface, as shown in Figure 2d—to produce a uniform film with a modifiable quality. Quality control is done by managing the evaporation rate, whether by adjusting the airflow over the substrate or heating the substrate to reach the boiling point of the solvent. A simple, environmentally friendly, vacuum free, low-cost deposition method and the capability to control film quality are some of the advantages offered by this method. Moreover, it is capable of overcoming performance degradation due to the presence of moisture and air [83]. This is caused by the slow crystallization rate that forms large-area grains that restricts the air and moisture permeability of the perovskite layer [84]. This method is suitable and stable and yields a great morphology for the deposited film.

#### 2.2.6. Slot-Die Coating

Slot-die coating is a solution processed deposition method that applies solution to flat substrates, such as glass or metal, and is highly scalable for large-scale area perovskite PVs, as shown in Figure 2e. An advantage to this method is less, or no, material wastage compared to spin-coating techniques. Moreover, it also produces a uniform and controllable film thickness by controlling the amount of material that is fed into the process. Hence, it can be used on a commercial scale for perovskite PV production [85].

#### 2.2.7. Ink-Jet Printing

Ink-jet printing is a common method used in the manufacturing of optoelectronic devices, field-effect transistors, and PVs. It is a non-contact technique that uses additive patterning. It is based on the selective ejection of ink from a chamber through a nozzle onto a substrate. A liquid droplet is ejected when an external bias is applied. This bias causes the chambers containing the liquid to contract, creating a shock wave in the liquid, and therefore ejection occurs, as shown in Figure 2f. The technique is considered fast, consumes less material, and can be used for large-scale production [86,87]. However, the main drawback is the possibility of a blocked nozzle because the used materials are poor solvents.

#### 2.2.8. Vapor-Based Method

The vapor-based method is an alternative method for fabricating perovskite PVs and it has better film uniformity compared to the solution-processing-based methods. This method is mostly used on an industrial scale for glazing, liquid crystal displays, and in the thin-film solar industry. The absorption efficiency of solar cells depends on the thickness of the perovskite layer. Thin layers absorb less sunlight, whereas thick films take a great deal of time to operate, as electrons and holes take significant time to reach their contacts. If the film is not uniform in the overall area, there will be direct contact with the electron transport material (ETM) and the hole transport material (HTM), resulting in a lower FF and V_oc_. Vapor-based deposition surpasses other techniques due to its ability to produce large-scale multi-stacked thin films with a uniform thickness. However, vapor-based deposition requires a vacuum to increase the mean free path between collisions to produce highly uniform and pure thin films. The vapor-based method is divided into two categories, physical and chemical.

#### 2.2.9. Chemical Vapor Deposition

**Figure 3:**
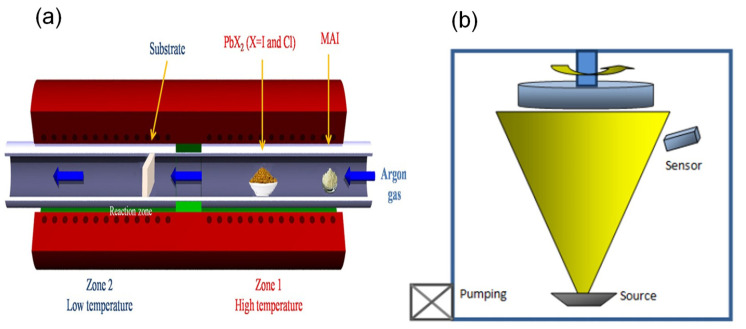
Type of vapor-based fabrication method: (**a**) chemical vapor deposition (CVD) [91]. Reproduced with permission from Ref. [91]. Copyright 2015 Scientific Reports. (**b**) Physical vapor deposition (PVD) [95]. Reproduced with permission from Ref. [95]. Copyright 2016 Scientific Reports.

Chemical vapor deposition (CVD) is a deposition method that produces highly scalable and pinhole-free large-scale perovskite PVs. A co-evaporation technique is used to deposit the perovskite layers with the help of two different precursors. The precursors are heated, mixed, and then moved to another substrate at a lower temperature, using a carrier gas (argon) to form highly uniform films, which are pinhole-free and have larger grain sizes and long carrier lifetimes, as shown in Figure 3a. CVD has been mostly deployed for fabricating perovskite layers to prevent the drawbacks of using low amounts of materials and the difficulty in controlling the flux deposition [91,92,93]. Moreover, while the use of CVD will produce a high material yield ratio and highly scalable for perovskite layer depositions [94], it requires a vacuum and uniform co-evaporation of the sample material, which is a challenge at an industrial scale.

#### 2.2.10. Physical Vapor Deposition

Physical vapor deposition (PVD) is a simple and non-reactive deposition process where a whole substrate surface area is covered, and the resulting layer has a high stability against moisture. Utilizing this method, a deposited film shows a uniform and smooth surface, with less defects and good crystallization layers. Figure 3b shows a single-source PVD for MAPbI_3_, where the temperature of the source is raised rapidly until the MAPbI_3_ evaporates without a chemical reaction and is then deposited on the substrate. The advantages of using PVD are the full surface coverage, great grain structure, high crystallization, scalability for mass production [95], and controllable film quality, thickness, and morphology, which makes PVD preferable to solution processing-based methods.

Table 1 shows various materials and their methods for HOIP PV fabrication, complete with the PCE values. With each material, for HOIP photovoltaics, improved, new and excellent perovskite materials with superior stability, light-absorption, charge mobility, and lifetimes were produced. The optimization of materials and structures is one of the keys to improving the photoelectric conversion efficiency [96]. The optimization of materials is shown in the work of Saliba et al. [67], via spin coating with different material compositions of Cs_0.05_(MA_0.17_FA_0.83_)_(0.95)_Pb(I_0.83_Br_0.17_)_3_ and Cs_0.1_(MA_0.17_FA_0.83_)_(0.90)_Pb(I_0.83_Br_0.17_)_3_ with the increase in the fill factor, from 74 to 77%. This is due to Cs_0.1_(MA_0.17_FA_0.83_)_(0.90)_Pb(I_0.83_Br_0.17_)_3_ having more uniform grains with each increase in Cs value, obtaining a better charge transport, which explains the higher fill factor value, which is consistent with works that used Cs_0.15_(MA_0.17_FA_0.83_)_(0.85)_Pb(I_0.83_Br_0.17_)_3_ as the perovskite material.

The optimization of device structure is shown in several works on the topic of fabricating MAPbI_3_ perovskite photovoltaics using different fabrication methods. Among MAPbI_3_ perovskite photovoltaic fabrication methods, spin coating shows the lowest PCE as the uniform structure is capable of obtaining a grain size of 100 nm [97]. Doctor blade and slot-die coating show a better PCE among all MAPbI_3_ perovskite photovoltaics with a more uniform grain size and thicknesses of 200 μm [98] and 5 μm [99], respectively, which are slightly better compared to spin coating due to the controllable fabrication methods.

**Table 1:** Various materials and methods for HOIP PV fabrications. Voc, Jsc, and FF are open-circuit voltage, short-circuit current density, and fill factor, respectively.

Coating Method	Material	Voc (V)	Jsc (mA cm^−2^)	FF (%)	Size (cm^2^)	PCE (%)	Ref.
Spin-coating	FAPbI_3_	1.06	24.7	77.5	~1	20.2	[100]
Spin-coating	Cs_0.05_(MA_0.17_FA_0.83_)_(0.95)_Pb(I_0.83_Br_0.17_)_3_	1.109	22.7	74.0	~1	18.6	[67]
Spin-coating	Cs_0.1_(MA_0.17_FA_0.83_)_(0.90)_Pb(I_0.83_Br_0.17_)_3_	1.13	22.0	77.0	~1	19.1	[67]
Ink-jet printing	Cs_0.1_(FA_0.83_MA_0.17_)_0.9_Pb(Br_0.17_I_0.83_)_3_	1.11	23.1	82	2.3	20.7	[101]
Spin-coating	Cs_0.15_(MA_0.17_FA_0.83_)_(0.85)_Pb(I_0.83_Br_0.17_)_3_	1.088	19.4	69.3	~1	14.6	[67]
Spin-coating	(FAPbI_3_)_0.95_(MAPbBr_3_)_0.05_	1.14	24.9	81	~0.094	23.2	[102]
Doctor blade	MAPbI_3_	1.12	22.6	81	0.075	20.3	[98]
Doctor blade	MAPbI_3_	1.10	22.7	81	0.08	20.2	[103]
Slot-die coating	MAPbI_3_	1.03	22.1	74	~232.3	16.8	[99]
Spin-coating	MAPbI_3_	1.08	20.7	68	0.16	15.2	[97]
Ink-jet printing	MAPbI_3_	1.08	22.66	76.2	0.04	18.6	[104]
Slot-die coating	MAPbI_3-x_Cl_x_	1.06	21.7	~78	0.06	18.0	[105]
Slot-die coating	MAPbI_3-x_Cl_x_	1.09	22.38	74.7	0.096	18.3	[106]
Spray coating	MAPbI_3-x_Cl_x_	1.10	21.4	77.6	0.08	18.3	[20]
Spin-coating	MAPb(I_0.85_Br_0.15_)_3_	1.07	21.5	68	0.076	15.4	[107]
Spray-coating	CsI_0.05_((FAPbI_3_)_0.85_(MAPbBr_3_)_0.15_)_0.95_	1.10	22.3	73	0.16	17.8	[19]
Screen printing	(AB)_2_(MA)_49_Pb_50_I_151_	0.94	23.4	71	0.8	15.6	[30]

Based on Table 1, it can be concluded that the best material for perovskite PV fabrication is (FAPbI_3_)_0.95_(MAPbBr_3_)_0.05_ [102]. Among the fabrication methods, the doctor blade method can be considered as the best method since it resulted in the highest PCE value. Such a method may improve the PCE of (FAPbI_3_)_0.95_(MAPbBr_3_)_0.05_, which already reaches 23.2% with spin coating [102].

## 3. Materials and Device Characterizations

This section describes the material characteristics that should be met for perovskite PVs, including the structural and morphological properties. XRD analysis can be used to detect a crystallite structure from the layer formation [108]. Based on synthesis conditions, there are three types of MAPbI_3_ structure. At T < 163 K, it is in an orthorhombic phase, at 163 K < T < 327.3 K, it is in a tetragonal phase and beyond 327.3 K, it will begin to form a cubic phase [109]. Substantial changes occur at 60 °C, as it shifts from the tetragonal to the cubic phase, with better grain sizes, based on the XRD results from several research studies, as multiple new peaks are present compared to the tetragonal phase [110,111,112].

The properties of HOIPs are also affected by organic monocation. Adding a bulky hydrophobic organic cation to the perovskite lattice can prevent moisture intrusion [113]. Because of this structure, the bandgap is large and tunable. Moreover, increasing the chain length of the organic monocation allows the prevention of oxidation of Sn^2+^ to Sn^4+^ [114].

Empirically, the structural stability of 3D perovskites can be predicted using the Goldschmidt tolerance factor, as the rule of thumb, for the ABX_3_ crystal structure, $t=\frac{\left(r_{a}+r_{x}\right)}{\sqrt{2\left(r_{b}+r_{x}\right)}}$, which is calculated based on the radii of the A, B and X ions. The ideal *t* value for a stable perovskite would be 0.8 < *t* < 1.0, so the organic monocation value has to be adjusted to obtain the necessary *t* value [115]. However, the perovskite ligand size can have an influence on this value [116].

**Figure 4:**
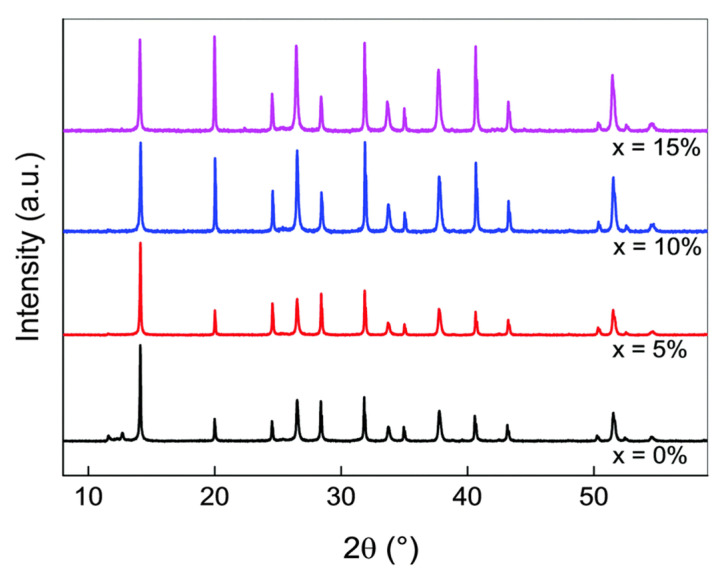
XRD characterization of Cs_x_M compounds. XRD spectra of perovskite with addition of Cs Cs_x_(MA_0.17_FA_0.83_)_(1−x)_Pb(I_0.83_Br_0.17_)_3_, abbreviated as Cs_x_M, where M stands for “mixed perovskite”. Cs_x_M with x = 0, 5, 10, 15%. Reproduced with permission from Ref. [67]. Copyright 2016 Energy and Environmental Science.

A key example to improve perovskite PV performance would be by using XRD techniques that will detect the best crystal structures in the formation of layers of a sample perovskite Cs_x_M, with x = 0, 5, 10, 15%, as shown in the XRD spectra of Figure 4. At about 14°, all Cs_x_M compositions with varying x values show the same perovskite peak. For Cs_0_M, there are small peaks observed at about 11.6° and 12.7° that represents the undesirable yellow non-perovskite phase (δ-FAPbI_3_) and the PbI_2_ that was used in its fabrication, respectively. As Cs amounts of 5, 10, 15% in the XRD spectra did not show these specific peaks, this would indicate that the mixed perovskite Cs_0_M shows an incomplete conversion of FA perovskite to the black-phase perovskite (α-FAPbI_3_).

**Figure 5:**
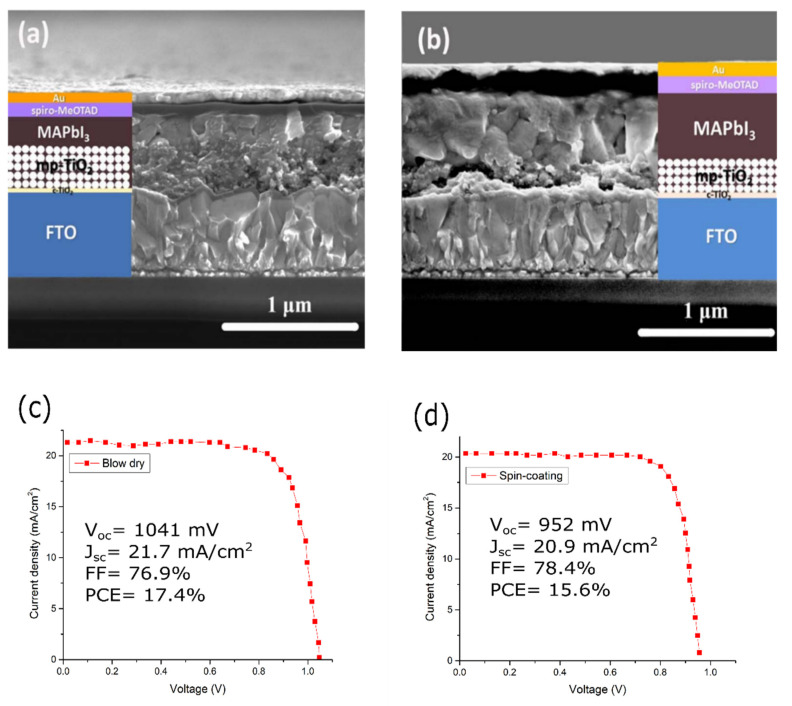
SEM images and J-V curve of the fabricated devices by using: (**a**,**c**) blow-dry and (**b**,**d**) spin-coating methods [117]. Reproduced with permission from Ref. [117]. Copyright 2017 Solar Energy Materials and Solar Cells.

Other than XRD characterization, with the different methods implemented for the fabrication of perovskite PVs, the resulting devices will also have different crystal structures relative to their fabrication methods [117]. The morphology of the perovskite is another factor to improve perovskite PV performance. Numerous work have been done on optimizing morphology by improving the fabrication methods [84,93] and by applying additional additives [118,119]. As shown in Figure 5a,b, different crystal grain structures were observed from using different fabrication methods (blow-dry and spin-coating).

The thicknesses of the layers differ between those fabricated using blow-drying and those using spin coating. For example, the thickness of the MAPbI_3_ and spiro-MeOTAD deposited by blow-drying is thinner than that fabricated by spin-coating. The blow-dried spiro-MeOTAD layer has been shown to be more compact and smaller than the spin-coated spiro-MeOTAD layer. Moreover, blow-dried MAPbI_3_ merged better with mp-TiO_2_ than the spin-coated MAPbI_3_, as shown in Figure 5c,d. As a result, devices with blow-dried MAPbI_3_ consistently achieve higher V_OC_, J_SC_ and PCE values than spin-coated ones.

Based on the work of Zhou et al. [120], the characteristics of the grain boundaries, grains, and material composition will have an effect on carrier transport, emissions, ionic diffusion, and performance. The simple crystal structures of the 3D halide PVs, such as MAPbI_3_ and FAPbI_3_, were determined using XRD or SEM analysis.

The presence of defects and grain boundaries in the PV film will have a detrimental effect on PV performance. Defect passivation is a strategy developed to enhance device efficiency and stability at the same time [121,122,123]. As shown in the work of Medjahed et al. [124], adding an additive in the presence of chlorine during the MAPbI_3_ synthesis has a positive advantage and impact on the growth and morphology (improvement on crystallite size and structure) of the obtained fabrication. Moreover, it also improves the electrical properties of the material, such as the electron diffusion length, carrier lifetime, and a high PCE value of 11.4%. It was also revealed, using in situ XRD, that the presence of two areas of MAPbI_3_ showed the presence of MAPbCl_3_. There were crystalline phases that could not be identified, but did not correspond to a chlorine-based intermediate phase, but rather the formation of MAPbCl_3_ in the perovskite formation from PbCl_2_. It can be concluded that MAPbI_3_ forms as annealing begins, MAPbCl_3_ disappears gradually, and that MAPbI_3_ decreases as the formation of PbI_2_ into MAPbI_3_ is completed, which corresponds with the work done by Saliba et al., who showed that XRD is able to analyze this information based on the patterns of the formation of the perovskite.

**Figure 6:**
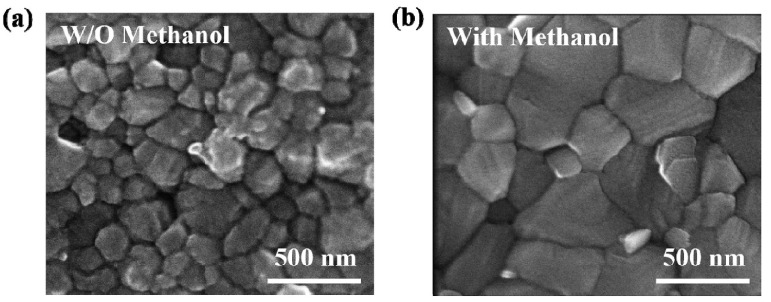
SEM images of the perovskite PV fabricated: (**a**) without and (**b**) with (5 vol%) methanol [125]. Reproduced with permission from Ref. [125]. Copyright 2019 Electrochimica Acta.

Feng et al. also demonstrated another additive enhancement to the morphology in a one-step solution-processed perovskite that utilizes methanol as shown in Figure 6 [125]. It was observed that, by adding a proper amount (5 vol%) of methanol solution to the perovskite (MAPbI_3_) precursor solution, the morphology, crystallization, optical and electrical properties of the perovskite layer were enhanced. SEM analysis was performed to investigate the morphology of the perovskite without methanol, and is shown in Figure 5a, and with (5 vol%) methanol solution, shown in Figure 5b. Under SEM, the perovskite film without methanol shows pinholes and grain boundaries. These pinholes and grain boundaries will impede charge transport, which easily induces the recombination between electron and hole, thus reducing the overall PV performance. However, the addition of methanol significantly enhances the morphology since it has rougher surface, larger grain sizes, and fewer grain boundaries compared to perovskite without methanol. Since the grain size in the vertical direction, as shown under SEM, is equivalent to the thickness of the perovskite, this indicates that the charge carriers can transport efficiently across the perovskite film and reach the electrodes before recombination occurs. The fabricated perovskite using methanol shows a higher PCE of 19.51% compared to the fabricated perovskite without methanol (16.53%). Moreover, it also has a better stability since it still shows a high PCE in the dark under ambient temperature for 30 days.

While the addition of additives may have improved the morphology of the HOIP, its lifetime and performance were not satisfactory for commercial and industrial applications. This will require further enhancements to the HOIP-like tandem structure to improve the overall lifetime and performance.

### 3.1. Perovskite Tandem Photovoltaics

Perovskite tandem PVs have been suggested as a method to improve the overall performance, stability, and lifetime of perovskite PVs. A perovskite tandem PV usually consist of a cell—either silicon, perovskite, or copper indium gallium selenide (CIGS)—overlaid by a perovskite PV [126], to increase the efficiencies beyond a single junction limit [127], without adding a substantial cost during production [128,129]. Typical single-junction PV cells do not make use of 67% of the solar energy they receive, because of the weak absorbance capabilities of the semiconductors. Semiconductors can only absorb photons with energy is above their bandgap energy (E_g_) and they generate energy equal to E_g_, where the rest of the energy are lost through thermalization as heat. This will severely affect the PCE of a PV because it corresponds to the V_oc_ and J_sc_ of the PVs. As one of solutions, tandem photovoltaics can address this problem [130].

Tandem PVs use stacks of materials with different bandgaps, where materials with larger bandgaps are put at the top of a cell and those with small bandgaps are at the bottom. High-energy photons are absorbed by the upper materials, while low-energy photons are not lost, in this case, but rather are absorbed by the lower stack materials, making use of most of the incident energy. One of the most known structures of tandem cells is the double-junction tandem device. They have two different configurations: two-terminal (2-T) and four-terminal (4-T) tandem, according to the stacking method used [130].

**Figure 7:**
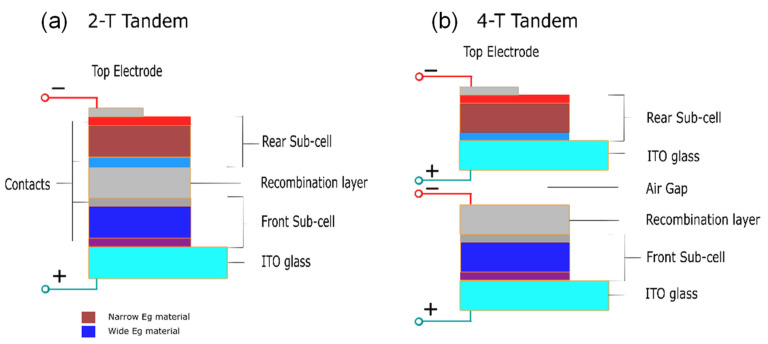
Schematic diagram of (**a**) 2-T and (**b**) 4-T tandem perovskite.

The 2-T tandem cells are synthesized by stacking a transparent front electrode, with a front cell and an opaque rear electrode, with the rear cell being one substrate where an interconnection layer (ICL) separates them, as shown in Figure 7a. The recombination of the photogenerated carriers from either sub-cell takes place in the ICL. On the other hand, 4-T tandem cells are made of two separate devices with two separate electrodes, linked together through a dichromatic mirror, as shown in Figure 7b. However, due to the additional electrodes, the optical loss will result in a more expensive cost compared to 2-T tandem PVs. Moreover, 2-T tandem PVs are cheaper to fabricate, though it is harder to fabricate 2-T or monolithic tandem PVs compared to 4-T tandem PVs for general applications [130].

Based on the values shown in Table 2, Voc, Jsc, and FF have similar in values with the 4-T Si/MAPbI_3_ configuration [131,132,133] and the values of Voc, Jsc, and FF in these works are about 1.1 V, 21 mA cm^−2^, and 75–80%, respectively. 

This occurs similarly in the 4-T CIGS/Cs_0.05_(MA_0.17_FA_0.83_)Pb_1.1_(I_0.83_Br_0.17_)_3_ configuration [138] and the 2-T CIGS/Cs_0.05_(MA_0.17_FA_0.83_)_0.95_Pb(I_0.83_Br_0.17_)_3_ configuration [33], since these works obtained similar values for Voc, Jsc, and FF of about 1.6–1.8 V, 17.0–19.2 mA cm^−2^, and 72.0–75.7%, respectively.

In comparison to the PCE values in Table 2, the 2-T configurations of CIGS/Cs_0.05_(MA_0.17_FA_0.83_)_0.95_Pb(I_0.83_Br_0.17_)_3_ [33,138] are better by 1.08-fold compared to those of the 4-T configurations. The 4-T configurations experience optical loss, which is found in the encapsulation and absorption in the transparent conductive electrode [139], which affects the PCE. The 2-T configurations experience series resistance loss in the large-area modules [140].

Further analysis is required to determine the increase in the PCE of the devices through development and improvements using tandem technologies. The configurations in Table 2 for tandem PVs refer to the materials in Table 1. The best performing PCE is from the Cs_0.05_(MA_0.17_FA_0.83_)_(0.95)_Pb(I_0.83_Br_0.17_)_3_ configuration in Table 1, and shows a PCE of 18.6% [81]; the best performing PCE from the 2-T Si/Cs_0.05_(FA_0.77_MA_0.23_)_0.95_Pb(I_0.77_Br_0.23_)_3_ configuration has a PCE of 29.3% [4] with an improvement achieved through tandem technologies using silicon and also through the use of an appropriate carbazole-based layer to efficiently extract the holes. Silicon was used as the bottom cell of the tandem since it has the properties of absorbing solar radiation from the near infrared region of the absorption spectrum. There is also another development in tandem PVs between Cs_0.05_(MA_0.17_FA_0.83_)_0.95_Pb(I_0.83_Br_0.17_)_3_ perovskite and CIGS, and the PCE value of the Cs_0.05_(MA_0.17_FA_0.83_)_0.95_Pb(I_0.83_Br_0.17_)_3_ tandem [33] increases by 1.25-fold compared to that of the intrinsic one [67].

The CIGS allows for a tunable bandgap, and it varies based on the temperature. A tunable bandgap was obtained from 1.1 to 2.3 eV by interchanging the cations, metals, and halides. These allow for the tandem PV to absorb photons that have an energy above the bandgap, so a higher bandgap will require a higher energy for the absorption spectrum. The best configurations for perovskite tandem PVs came from silicon and CIGS. In fact, another perovskite–perovskite tandem PV has been recorded, with a high PCE of 24.4% [141] and there are few reports presently beating this record. Therefore, the best reported tandem perovskite photovoltaic belongs to the 2-T Si/Cs_0.05_(FA_0.77_MA_0.23_)_0.95_Pb(I_0.77_Br_0.23_)_3_ configuration with a PCE of 29.3% [4].

For further commercialization of perovskite PVs, large area modules should be developed since the previous studies were conducted for a small area (1 cm^2^). This development needs to be carried out to determine if there is any improvement in the crystal grain structure and PCE value when the module size is increased.

### 3.2. Large Scale Modules

Several issues need to be addressed for further perovskite PV commercialization: (i) a thin-film deposition method that is scalable and reproducible; (ii) high stability and long lifetime; and (iii) low or less toxic materials for large-area devices.

The rapid increment in PCE performance for perovskite PV has developed at a quick pace compared to other solar technologies. However, several high PCE values have been obtained with very small areas (~1.0 cm^2^). To fabricate a large area with a high-quality perovskite (good uniformity and few structural defects), several deposition methods have been developed, such as spin-coating, slot-die coating, doctor blade, and vacuum deposition. In 2015, Chen et al. [142] fabricated perovskite using one-step spin-coating with an active area of 1.02 cm^2^ and obtained a PCE of 15%. In 2016, Qiu et al. [143] fabricated a large-area perovskite with an active area of 1 cm^2^ and obtained a PCE of 13.6%.

While spin-coating is widely used in the deposition of small-area thin films in laboratories, it might not be suitable for industrial large-area fabrications. Spin-coated films are not uniform throughout the area [144], require a large amount of solution mixture of materials, and there is significant wasted solvent, which increases the cost of fabrication. Its performance also reduced due to the increase in series resistance and the decrease in film quality [145,146]. As in the case of Hossain et al. [147], since spin-coating is not suitable for the fabrication of perovskite solar cell on a silicon solar cell, so some methods have to be replaced by PVD or CVD.

In 2018, Zheng et al. [148] were able to achieve 21.8% PCE using CVD on a 16 cm^2^ monolithic (FAPbI_3_)_0.83_(MAPbBr_3_)_0.17_ perovskite–silicon tandem PVs. The final product of the PVs has an enhanced V_oc_ with a J_sc_ that ranged from 15.6 to 16.2 mA/cm^2^ and an FF of 78%. The supporting information regarding further improvements that could be achieved for this device would be the spiro-OMeTAD stack being replaced with a high refractive index inorganic hole transport layer to eliminate unnecessary wavelength absorptions. To improve it for commercial use in larger area of 6″ × 6″, the PDMS layer can be replaced with thin glass and the spin-coating process can be replaced with something less expensive, such as the doctor blade or spray-coating methods, which will be discussed in further works.

**Figure 8:**
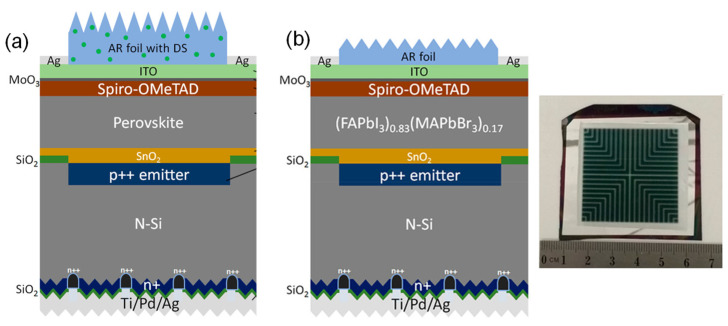
(**a**) Schematic of perovskite–silicon tandem homojunction photovoltaic with downshifting AR PDMS layer. From Ref. [149]. Copyright 2019 ACS Energy Letters. (**b**) Schematic of monolithic (FAPbI_3_)_0.83_(MAPbBr_3_)_0.17_ perovskite/rear-textured-homo-junction-silicon tandem photovoltaics. Reproduced with permission from Ref. [148]. Copyright 2018 ACS Energy Letters.

After conducting their previous work, in 2019, Zheng et al. [149] showed another improvement that increased the PCE value. They decided to use micron green-emitting (Ba,Sr)_2_SiO_4_:Eu^2+^ phosphor—a cheap material that is commercially available and is mostly used in light-emitting diodes—as an antireflective down shifting material on PDMS that acts as the hydrophobic layer of the silicon–perovskite tandem PVs with an area of 4 cm^2^, which achieved a PCE of 23%. SEM images of the (Ba,Sr)_2_SiO_4_:Eu^2+^ sample on PDMS showed a high-quality crystal size of about 10–20 μm. Moreover, the results of energy-dispersive X-ray spectroscopy (EDX), not only confirmed the homogeneous distributions of Sr, Ba, Si, and O within the specimen, but were also able to detect the Eu content. Figure 8 shows schematic diagrams of the (a) previous and (b) current large-scale modules of perovskite photovoltaics.

After further investigation, with a higher concentration of (Ba,Sr)_2_SiO_4_:Eu^2+^ phosphor on top of the perovskite–silicon PV, with initial conditions of J_sc_ of 14.1 mA/cm^2^, V_oc_ of 1.73 V, FF of 82%, and a PCE of 20.1%, the result shows that, while the open-circuit voltage remains unchanged or at a constant level, there was an improvement in J_sc_ that was caused by the increased broad absorption of the cell with a reduced front reflection and increased light trapping. Eventually the best choice was obtained and had an area of 4 cm^2^ with conditions of J_sc_ of 16.4 mA/cm^2^, V_oc_ of 1.73 V, FF of 81%, and a PCE of 23%.

Although works on monolithic perovskite–silicon tandem PVs showed progress in terms of PCE values (about 22–23%), there are several issues that need to be solved for further large-scale fabrication. These issues include stability improvements, the degradation rate, commercialization costs, and the use of environmentally friendly materials.

### 3.3. Improvements to Perovskite Material and Its Tandem Structures

A recent investigation, conducted by Green et al. [1] on perovskite–silicon tandem PVs, states that a high PCE value of 29.15% had been achieved with an area of 1.060 cm^2^. Though it is a small area, in the future, people will continue to develop perovskite–silicon tandem photovoltaics, which will progress further so that it is possible to achieve 4 cm^2^ perovskite–silicon tandem photovoltaic with an efficiency of 29.15%, if there is a breakthrough on how to further increase the broad absorption of a cell with reduced reflection but while increase the light trapping.

Furthermore, since it is difficult to deposit a large area of continuous perovskite film using the previously described traditional methods, other methods should be improved to prepare high-quality and large-area perovskite PVs for commercial production in the future. Moreover, from the perspective of green energy, the Pb employed in perovskite PVs is highly toxic, which will hinder the industrial promotion and development of perovskite PVs. Therefore, it is necessary to find a low-toxicity or non-toxic material to replace Pb in the future [96].

Realistically, the halide of Pb is 10 times more dangerous than the Pb that already exists on Earth [150,151,152,153]. Several lines of research indicate that contamination due to leaks of lead ions into soil and water resources is a permanent affliction and generates harmful effects on humans, animals, and plant survivability [154,155,156,157,158,159,160,161,162,163]. To decrease and reduce its toxicity level, Pb-free [164,165,166] (or at the very least less Pb content) perovskite–silicon PVs have been researched using a safer option by mixing chalcogen and halogen anions [167] or using tin or (Sn)-based perovskite PVs [168,169,170]. Recently, it was determined that there is an all-perovskite Pb–Sn tandem photovoltaic with a PCE of 26.4% [171], showing a value that potentially exceeds that of the best-performing single-junction perovskite solar cells, which are capable of retaining more than 90% of their initial performance after 600 h of operation at maximum power under one-sun illumination under ambient conditions. Recent research [172], developed a low-cost device made of sulfonic acid-based lead-adsorbing resin, which freezes lead ions into a scaffold and prevents their leakage when the perovskites are exposed to rainfall. This new device does not affect efficiency or scaling and the structure can be scaled up to 60.8 cm^2^.

However, Sn-based perovskite PVs have lower efficiencies and faster degradation than Pb-based perovskite PVs due to their phase fluctuations and they easily oxidize from Sn^2+^ to Sn^4+^. In the case of Sn-based perovskite PVs being unable to breach the efficiency limit of Pb-based perovskite PVs, new approaches had been created to prevent Pb leakage in perovskite PVs by trapping Pb with cation-exchange resins that are abundant in Ca^2+^ and Mg^2+^ [173].

In addition, the cost barrier for perovskite–silicon cells was identified as being due to expensive organic charge transport materials, such as spiro-OMeTAD, PTAA, and PC_60_BM [174,175,176,177]. The cost of organic charge transport, especially for one with a higher quality that can yield a better performance, is very expensive due to the intricate nature of the fabrication steps, in addition to the additional costs that might be derived from better or higher purity in terms of the formation of crystal grains. As an alternative to using organic charge transport materials, inorganic charge transport materials, such as NiO, CuSCN, SnO_2_, and Nb_2_O_5_, which are much cheaper in comparison to organic charge transport materials, have been successfully developed for some perovskite PVs [101,178,179,180].

Further improvements concern the degradation of the perovskite PVs. PVs are always exposed to various degradation sources, such as humidity, oxygen, heat, and ultraviolet light. To ensure the lifetime of perovskite–silicon tandem PVs, their stability needs to be tested and improved against degradation. Among perovskite PVs, methyl ammonium lead triiodide is easily degraded by humidity and heat, in comparison to perovskite PVs that are based on FAPbI_3_ [60] or CsPbI_3_ [181], which proved to have a higher resistance to thermal decomposition—which can be solved by mixing the cations and the halogen anions, which improves the thermal and crystal structure stability. As an example of mixing halide anions, bromide-based perovskite PVs show better resistance to degradation under humidity and heat when compared to iodide-based perovskite PVs as they developed a 2D or 3D hetero-structure in the perovskite PVs [182,183,184].

**Figure 9:**
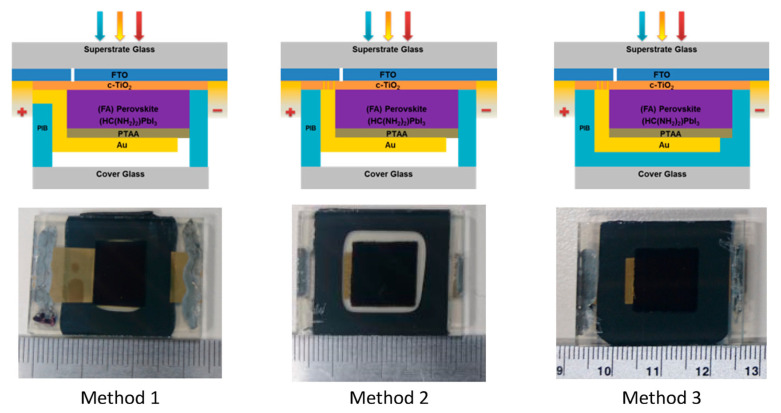
Cross-sectional schematics with the photographs of perovskite solar cells encapsulated by three different methods [188]. From Ref. [188]. Copyright 2017 ACS Applied Materials and Interfaces.

It is also suggested that organic charge transport materials, such as spiro-OMeTAD, PTAA, and PC_60_BM, are easily disintegrated by humidity and oxygen, so they require a higher level of encapsulation to prevent the elements from disintegrating the organic charge transport materials. An alternative solution would be to use inorganic charge transport materials, which are beneficial in terms of their stability due to their basic properties. In addition, a densely formed inorganic layer can act as a diffusion barrier to prevent organics or iodine species escaping from the lattice and reacting with the top metal electrode [185,186]. It has been reported in the literature that a semi-transparent perovskite PV with a dense charge transport layer and a transparent electrode endured through thermal cycling, damp heat, and UV stress tests [185,186,187]. Moreover, low-temperature, glass–glass encapsulation techniques, using high-performance polyisobutylene (PIB) on planar perovskite solar cells, have been reported using three different electrical configurations and methods are as shown in Figure 9 [188]. In method 1, PIB is put on the top of a thin gold film that acts as a positive electrical feedthrough for the cell. In methods 2 and 3, the FTO layer provides the electrical feedthroughs. It is worth noting that the PIB in methods 1 and 2 plays the role of an edge seal, but it blankets the entire area under the glass in method 3.

Lead-free PVs are also being investigated as potential improvements to PVs [157,164], as these same materials were investigated as scintillators [114,189]. Several improvements regarding their material quality, degradation have been considered, as have techniques to fabricate them. To further improve the commercialization rate in the future, certain costs that are more expensive, such as organic charge transport materials, must be replaced. A better alternative would be the use of inorganic charge transport materials to reduce costs. While these methods will reduce the cost for commercialization, the most harmful content, which is the lead, will need to be replaced, reduced, or they will need to be made using Pb-free methods, discussed above, to reduce harm to the environment.

Several improvements have been achieved for perovskite–silicon tandem PVs, such as a longer lifetime, lower cost, and less-harmful chemicals being used. These achievements have inspired some companies to produce and commercialize perovskite–silicon tandem PVs.

### 3.4. Commercialization

For future commercialization, although there are several challenges to be faced in fabricating perovskite PVs, some companies are in the early stages of developing perovskite PVs, e.g., Saule technologies [190] and Quantum Solution [191]. These developments are driven by perovskite’s wide bandgap, low-cost, and simple fabrication methods. For Saule technologies, they launched the first industrial production line of solar panels in May 2021 in Poland, based on groundbreaking perovskite technology. They are making sheets of solar panels using a novel inkjet printing procedure invented by the founder, Olga Malinkiewicz [190].

However, the long-term performance of such a PV is an issue to pursue. As an example, the longest lifetime for the prototype was 6000 h under continuous one-sun illumination before degrading beyond 80% of its initial performance [192]. Interfacial recombination can be a severely degrading performance issue, but some methods are used to inhibit this. In [193], a 2D octyl-diammonium lead iodide interlayer was used to decrease recombination losses and obtain a PCE of 22.27% in tandem solar cells. A 2D/3D perovskite interface in [194] suppressed interface losses with a PCE ≈ 21%. As an alternative, perovskite–silicon tandem PV has a better lifetime of 300 h of operation, and retained 95% of its initial efficiency without encapsulant, and performs at a PCE of 29.3% in comparison to the theoretical limit of 29% for the silicon PV [195]. Therefore, the perovskite–silicon tandem PV is a prospective option for future commercialization; particularly, OxfordPV has pioneered producing their heterojunction silicon PVs to enhance their PV cells [196].

### 3.5. Summary

**Figure 10:**
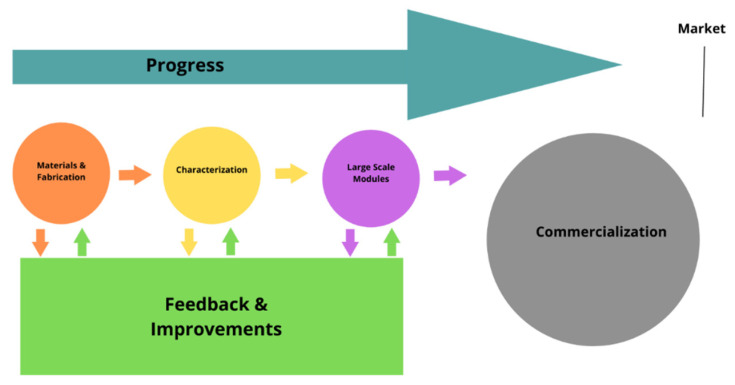
Scheme of the progress of perovskite tandem PVs.

HOIPs have come a long way since their predecessors, with a current standing performance (or PCE) of about 29.3%. In this review, we highlighted the progress of HOIP materials and large-area fabrication techniques, in detail, and provided several comparisons between the techniques, and materials used in the fabrication of solar cells through their power conversion efficiencies. The fabrication techniques were also covered along with the advantages and disadvantages of using certain materials, which further enhance the properties of perovskite–silicon tandem solar cells; as shown using SEM. A scheme of this is shown in Figure 10, and it shows how far the progress of research towards commercialization and market must go. Although there has been feedback and improvements for materials, fabrication, characterization, and large-scale modules, commercialization is just starting. To enter the market of PVs, the other aspects, such as the total cost (although the material is cheap enough) and the aging of perovskite materials relative to those made using silicon, need to be addressed. Advances in other materials for optoelectronic devices such as graphene, MoS_2_ and compound semiconductors [197,198,199,200,201,202,203,204,205,206,207,208,209,210,211,212] are having a huge impact and benefitting the progress in the field of PVs either through the process techniques, or through the marketization strategies. In the end, the perovskite–silicon tandem solar cells have been introduced as the next generation of PVs and will replace the current technology of silicon PVs (in comparison of electric costs) [6].

## Figures and Tables

**Table 2:** Some reports of perovskite tandem PVs. Voc, Jsc, and FF are open-circuit voltage, short-circuit current density, and fill factor, respectively.

Type	Tandem	Perovskite Material	Voc (V)	Jsc (mA cm^−2^)	FF (%)	Size (cm^2^)	PCE (%)	Ref.
4-T	Si	MAPbI_3_	1.08	20.6	74.1	0.075	23.0	[131]
4-T	Si	MAPbI_3_	1.098	21.0	74.1	1.10	26.7	[132]
4-T	Si	MAPbI_3_	1.156	19.8	79.9	1.00	27.0	[133]
2-T	Si	MAPbI_3_	1.69	15.9	77.6	0.17	21.2	[134]
2-T	Si	Cs_0.05_(FA_0.77_MA_0.23_)_0.95_Pb(I_0.77_Br_0.23_)_3_	1.87	19.37	79.9	1.06	29.3	[4]
2-T	Si	Cs_0.05_(MA_0.17_FA_0.83_)_0.95_Pb(I_0.83_Br_0.17_)_3_	1.792	19.02	74.6	1.088	25.2	[135]
2-T	Si	Cs_0.15_(MA_0.17_FA_0.83_)_0.85_Pb(I_0.7_Br_0.3_)_3_	1.80	17.8	79.4	~1.00	25.4	[136]
4-T	CIGS	Cs_0.09_FA_0.77_MA_0.14_Pb(I_0.86_Br_0.14_)_3_	1.77	17.3	73.1	0.04	22.4	[137]
4-T	CIGS	Cs_0.05_(MA_0.17_FA_0.83_)Pb_1.1_(I_0.83_Br_0.17_)_3_	1.59	18.0	75.7	0.78	21.6	[138]
2-T	CIGS	Cs_0.05_(MA_0.17_FA_0.83_)_0.95_Pb(I_0.83_Br_0.17_)_3_	1.68	19.2	71.9	1.03	23.3	[33]
